# Effect of early tranexamic acid treatment on fatigue in patients with mild traumatic brain injury: data from the CRASH-3 clinical trial

**DOI:** 10.12688/wellcomeopenres.17421.2

**Published:** 2024-09-03

**Authors:** Raoul Mansukhani, Antonio Belli, Amy Brenner, Rizwana Chaudhri, Lauren Frimley, Sabariah Faizah Jamaluddin, Rashid Jooma, Haleema Shakur-Still, Temitayo Shokunbi, Ian Roberts

**Affiliations:** 1 Clinical Trials Unit, London School of Hygiene & Tropical Medicine, Keppel Street, London, WC1E 7HT, UK; 2 College of Medical and Dental Sciences, University of Birmingham, Birmingham, UK; 3 Global Institute of Human Development, Shifa Tameer-e-Millat University, Rawalpindi, Pakistan; 4 Department of Emergency Medicine, Faculty of Medicine, Universiti Teknologi MARA, Sungai Buloh Campus, Malaysia; 5 Department of Surgery, Aga Khan University Hospital, Karachi, 74800, Pakistan; 6 Department of Neurological Surgery, University College Hospital, Ibadan, Oyo State, PMB 5116, Nigeria

**Keywords:** Traumatic Brain Injury, Ttranexamic Acid, Fatigue, CRASH-3 trial, Randomised Controlled Trial, IntracranialHaemorrhage

## Abstract

**Background:**

Each year world-wide about 65 million people sustain a mild traumatic brain injury (mTBI). Fatigue is a common and distressing symptom after mTBI. We examine the effect of tranexamic acid (TXA) on fatigue in patients with mTBI using data from the CRASH-3 trial.

**Methods:**

The CRASH-3 trial randomised 9,202 patients with traumatic brain injury and no significant extracranial bleeding to receive TXA or placebo within 3 hours of injury. The primary outcome was death from head injury within 28 days of injury. The methods and results are presented elsewhere. Fatigue was recorded as “None”, “Moderate” or “Extreme.” This study examines the effect of TXA on extreme fatigue in the 2,632 patients with mTBI (Glasgow Coma Scale [GCS] score≥13). Our analyses were not prespecified.

**Results:**

Our study primary outcome, extreme fatigue, was reported for 10 (0.8%) of 1,328 patients receiving TXA and 19 (1.5%) of 1,288 patients receiving placebo (risk ratio [RR]=0.51, 95% confidence interval [CI] 0.24-1.09). Death within 28 days of injury was reported for 34 (2.6%) of 1,328 patients receiving TXA versus 47 (3.6%) of 1,288 patients receiving placebo (RR=0.70, 95% CI 0.45-1.08). Among patients allocated to TXA, 44 (3.3%) patients either died or reported extreme fatigue versus 66 (5.1%) patients among those allocated to placebo (RR=0.65, 95% CI 0.44-0.94). This composite outcome is disproportionately influenced by deaths which account for 74% (81 from 110) of events.

**Conclusions:**

We found no evidence that tranexamic acid reduces fatigue in patients with mTBI. Given, 1) our analyses were not prespecified, 2) our outcome measure is not based on a validated fatigue severity scale, and 3) TBI patients can suffer from hospital-induced delirium, which hinders clinician assessment, these results need to be replicated in another study.

**Registration:**

ISRCTN (ISRCTN15088122, 19/07/2011), ClinicalTrials.gov (NCT01402882, 26/07/2011), EudraCT (2011-003669-14, 25/07/2011), Pan African Clinical Trial Registry (PACTR20121000441277, 30/10/2012).

## Introduction

Each year, world-wide about 65 million people experience a mild traumatic brain injury (mTBI) ^1^. The most common causes are falls and road traffic crashes ^2^. With an increasing and ageing world population the number of people suffering mTBI is expected to rise.

Tranexamic acid reduces bleeding by inhibiting blood clot breakdown. The CRASH-2 trial ^3^ showed that giving tranexamic acid within 3 hours of injury reduces deaths due to bleeding in trauma patients. This raised the hope that tranexamic acid might reduce traumatic brain injury (TBI) deaths. Intracranial bleeding is common after TBI and causes death and disability. The CRASH-3 trial ^4^ was a large randomised trial of the effect of tranexamic acid on death and disability in patients with TBI. The primary outcome was head injury death. The results were published in 2019. The risk of head injury death was 12.5% in the TXA group versus 14.0% in the placebo group (risk ratio [RR] = 0.89, 95% confidence interval [CI] 0.80–1.00). The reduction was greater in patients with mild and moderate head injury (RR = 0.78, 95% CI 0.64–0.95) than in severe head injury (RR = 0.99, 95% CI 0.91–1.07). The effect of tranexamic acid on disability was assessed by comparing the Disability Rating Scale (DRS) ^5^ score in the tranexamic acid and placebo group. The mean DRS scores were similar in the tranexamic acid and placebo groups.

Fatigue is defined as overwhelming tiredness not relieved by sleep or rest and is a common and distressing symptom in patients with mTBI ^6^. Over one third of mTBI patients have fatigue six months after injury ^6^. TBI related fatigue has a detrimental effect on patients’ quality of life ^7, 8^ and TBI patents who report fatigue are at increased risk of anxiety and depression ^9– 11^. When planning the CRASH-3 trial, we discussed the proposed outcome measures with patient representatives who specifically requested that fatigue was included as an outcome.

Intracranial bleeding appears to increase the risk of fatigue. A recent prospective cohort study ^12^ found that mTBI patients with intracranial bleeding on computerised tomography (CT) or magnetic resonance imaging (MRI) scans were significantly more likely to experience fatigue compared to those without intracranial bleeding. Early tranexamic acid treatment may reduce intracranial bleeding in TBI patients. It therefore seems plausible that timely tranexamic acid treatment might reduce fatigue in mTBI patients. We examine the effect of tranexamic acid on fatigue in mTBI patients in the CRASH-3 trial.

## Methods

The CRASH-3 trial is registered at ISRCTN (ISRCTN15088122), ClinicalTrials.gov (NCT01402882), EudraCT (2011-003669-14), and the Pan African Clinical Trial Registry (PACTR20121000441277). This article is reported in line with the Consolidated Standards of Reporting Trials (CONSORT) guidelines ^13^.

The background to the CRASH-3 trial, the methods, baseline characteristics and main results were previously reported ^4, 14, 15^. Briefly, CRASH-3 trial participants were adults with TBI, who had a Glasgow Coma Scale (GCS) score ≤ 12 or any intracranial bleeding on CT scan and no significant extracranial bleeding. Patients were recruited from 175 hospitals in 20 countries. The primary outcome was head injury related in hospital death within 28 days of injury in patients treated within 3 hours of injury. Between July 2012 and January 2019, 12,737 patients with TBI were randomly allocated to receive tranexamic acid or placebo, of whom 9,202 patients were treated within 3 hours. This analysis focusses on the 2,632 mTBI patients who had a GCS score of between 13 and 15 and intracranial bleeding on CT scan who were treated within 3 hours of injury.

The trial treatment was 1g of tranexamic acid administered intravenously over 10 minutes followed by a 1g maintenance dose administered intravenously over 8 hours. Patients were allocated a treatment pack identifiable by a unique number. These unique numbers (randomisation codes) were prepared by an independent statistician from Sealed Envelope (UK) Ltd. Packs were equally likely to contain tranexamic acid or placebo. Once patients had been deemed suitable for trial entry, and baseline details obtained, they were designated the lowest numbered treatment pack remaining from a box of 8 packs. Each pack contained four ampoules of either 500g of tranexamic acid or placebo (0.9% sodium chloride), a 100ml bag of 0.9% sodium chloride (to be used for infusing the loading dose), a syringe with needle, stickers containing the randomisation number (to be attached to patient records, trial forms and saline bags) and a document of instructions for clinicians. Tranexamic acid and placebo treatment packs, labels and ampoules were identical. Patients, clinicians and trial coordinating centre staff were blinded to treatment allocation. Once a patient had been designated a treatment pack, providing the ampoules contained in that pack were not broken, they were considered to be randomised into the trial.

Fatigue was assessed by a clinician and recorded as “None”, “Moderate” or “Extreme”. Patient fatigue was recorded either at discharge or in hospital after 28 days of injury if the patient had not already been discharged. The statistical analysis plan ^15^ prespecified that we would report the effect of tranexamic acid on fatigue by estimating the risk ratio of being in the extreme fatigue category. We considered that the extreme category of fatigue would be less susceptible to misclassification bias and a better measure of the disabling patient fatigue that many mTBI patients experience.

We selected mTBI patients for this study motivated by a recent cohort study ^12^ which reported an association between intracranial findings on a CT or MRI scan and fatigue in mTBI patients. Severely injured patients are likely to suffer the effects of pathophysiological processes other than intracranial bleeding which tranexamic acid is unlikely to affect. Deaths and fatigue caused by these processes are likely to bias our treatment effect estimates towards the null.

We report risk ratios and 95% confidence intervals for the effect of early tranexamic acid treatment on extreme fatigue which is our study primary outcome. As a secondary outcome we report death within 28 days of injury. As an additional secondary outcome, we report the composite outcome “death or extreme fatigue” to highlight the potential benefits early tranexamic acid treatment may provide mTBI patients. This composite outcome is consistent with the International Council for Harmonisation of Technical Requirements for Pharmaceuticals for Human Use (ICH) E9 guidelines’ "Composite strategy" for handling intercurrent events ^16^. Patients with missing outcome data were excluded from our analysis. All analyses were performed in R version 4.1.1.

### Ethical approval and consent to participate

Most patients with TBI are unable to provide informed consent to participate in a clinical trial due to the nature of their injury. In the CRASH-3 trial, consent was sought from the patient’s relative or a legal representative unless no such representative was available, in which case the study proceeded with the agreement of two clinicians. If the patient regained capacity, they were told about the trial and written consent was sought to continue participation. If either the patient or their representative declined consent, participation was stopped. If patients were included in the trial but did not regain capacity, consent was sought from a relative or legal representative. We adhered to the requirements of the local and national ethics committees. The trial received a favourable opinion by London School of Hygiene & Tropical Medicine observational/interventions research ethics committee on 17 November 2011 (LSHTM ethics ref: 6060). 

## Results

**Figure 1.**
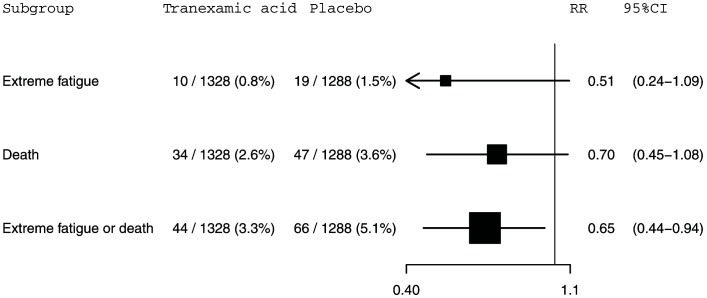
The effect of tranexamic acid on extreme fatigue, death within 28 days of injury and extreme fatigue or death within 28 days of injury for CRASH-3 trial participants with mild traumatic brain injury who were randomised within 3 hours of injury. N=2616. RR=risk ratio; CI=confidence interval.

The CRASH-3 trial randomised 2,632 patients with a GCS 13-15 to receive tranexamic acid or placebo within 3 hours of injury. Outcome data were collected for 2,616 patients of whom 1,328 received tranexamic acid and 1,288 received placebo ( Figure 1). Baseline characteristics of the trial participants are shown in Table 1.

**Table 1.** Baseline characteristics before randomisation for CRASH-3 trial participants with mild traumatic brain injury who were randomised within 3 hours of injury. TXA=tranexamic acid; SD=standard deviation.

	TXA (N=1335)		Placebo (N=1297)	
	n	%	n	%
**Sex**				
Men	1039	77.8%	974	75.1%
Woman	296	22.2%	323	24.9%
**Age at randomisation (years)**				
Mean (SD)	44.4	20.3	45.3	20.0
16–24	271	20.3%	235	18.1%
25–34	259	19.4%	236	18.2%
35–44	194	14.5%	211	16.3%
45–54	189	14.2%	195	15.0%
55+	422	31.6%	420	32.4%
**Time since injury (h)**				
Mean (SD)	2.0	0.7	2.0	0.7
≤1	184	13.8%	199	15.3%
1–2	559	41.9%	543	41.9%
2–3	592	44.3%	555	42.8%
**Systolic blood pressure (mm Hg)**				
0–89	11	0.8%	7	0.5%
90–119	408	30.6%	394	30.4%
120–139	492	36.9%	488	37.7%
140+	421	31.5%	405	31.2%
Unknown	3	0.2%	3	0.2%
**Glasgow Coma Scale score**				
13	297	22.2%	312	24.0%
14	526	39.4%	458	35.3%
15	484	36.3%	492	38.0%
Unknown	28	2.1%	35	2.7%
**Pupil reaction**				
Both React	1288	96.5%	1249	96.3%
One Reacts	33	2.5%	28	2.2%
None React	4	0.3%	3	0.2%
Unable to assess	10	0.7%	17	1.3%

**Figure 2.**
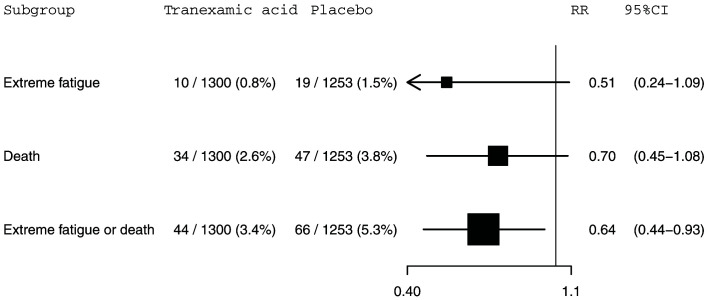
The effect of tranexamic acid on extreme fatigue, death within 28 days of injury and extreme fatigue or death within 28 days of injury for CRASH-3 trial participants with mild traumatic brain injury excluding patients with a missing GCS score who were randomised within 3 hours of injury. N=2553. RR=risk ratio; CI=confidence interval.

A total of 29 (1.1%) patients had extreme fatigue and 81 (3.1%) patients died within 28 days of injury. There were 110 (4.2%) patients who either died or experienced extreme fatigue. The mean age (standard deviation [SD]) of study participants was 45 (20) years. The mean (SD) age of participants with extreme fatigue was 61 (20) years. Extreme fatigue was reported for 6 (1.0%) of 618 women and 23 (1.2%) of 1998 men. Extreme fatigue was reported for 8 (0.5%) of 1687 patients in low to middle income countries and 21 (2.3%) of 929 patients in high income countries. The effects of tranexamic acid on extreme fatigue, death within 28 days of injury, and the composite outcome of extreme fatigue or death are shown in Figure 1. Extreme fatigue was reported for 10 (0.8%) out of 1,328 patients in the tranexamic acid group and 19 (1.5%) out of 1,288 patients in the placebo group (RR=0.51, 95% CI: 0.24-1.09). For death within 28 days of injury, 34 (2.6%) out of 1,328 patients died in the tranexamic acid group versus 47 (3.6%) out of 1,288 patients from the placebo group (RR=0.70, 95% CI: 0.45-1.08). The risk of the composite outcome of extreme fatigue or death was reduced by 35% in patients treated with tranexamic acid. There were 44 (3.3%) patients with this outcome among the 1,328 patients allocated to the tranexamic acid group compared with 66 (5.1%) patients among the 1,288 patients allocated to the placebo group (RR=0.65, 95% CI: 0.44-0.94). This composite outcome is disproportionately influenced by deaths which account for 74% (81 from 110) of events. Excluding the 63 patients with a missing GCS score from our analysis left these effect estimates and confidence intervals ( Figure 2) almost entirely unchanged.

## Discussion

Our study found no evidence that tranexamic acid reduced fatigue in patients with mTBI. Our analysis was not prespecified, the precision of the estimates was low and our outcome measure is not based on a validated fatigue scale. In addition, TBI patients are prone to hospital-induced delirium which will hinder clinician assessment of fatigue. A larger trial is needed to test the hypothesis that early tranexamic acid treatment reduces fatigue in mTBI patients.

Ours is the first trial to examine the effect of tranexamic acid on fatigue in mild TBI patients. The study was randomised and placebo controlled with a large sample size and minimal loss to follow up. However, our study has important limitations. Although we planned to examine the impact of TXA treatment on extreme fatigue, the focus on mild TBI was not pre-specified but stimulated by the observation from the CRASH-3 intracranial bleeding mechanistic study ^17^ that patients with less severe TBI may have less intracranial bleeding at baseline and so there is more potential to prevent bleeding. Patients with more severe TBI either have extensive intracranial bleeding at baseline or other intracranial pathologies that are not affected by TXA. On the other hand, because clinicians underestimated time to treatment ^18^ some mild TBI patients may have been treated beyond 3 hours of injury with reduced potential to prevent bleeding. Inaccuracy in time to treatment may have biased the estimated treatment effects of tranexamic acid towards the null. The CRASH-3 trial assessed fatigue via a three-point scale which hasn’t been validated. We acknowledge this is a significant limitation with our study. Ideally, fatigue would be measured using a validated scoring system such as the 9 item Fatigue Severity Scale (FSS). Other studies have found that around 30% of all mTBI patients experience fatigue ^12, 19– 22^. We cannot rule out clinicians underreporting extreme fatigue. However, this misclassification of our outcome variable would be expected to bias our results towards the null. Our study primary outcome extreme fatigue is measured at a single timepoint. This measure is an imperfect proxy for the disabling long-term fatigue many mTBI patients experience. Some patients with our outcome will eventually recover while others without the outcome will later experience fatigue. 

Intracranial bleeding in mTBI patients is associated with an increased risk of fatigue. Saksvik ^12^ used data from the Trondheim mild traumatic brain injury [mTBI] follow-up study to compare fatigue in patients with complicated (intracranial findings on a CT or MRI scan) and uncomplicated mTBI. Fatigue was assessed using the FSS questionnaire ^19, 23^. Patients were monitored for fatigue at 2 weeks, 3 months and 12 months. The percentage of patients with fatigue was higher at every time point for the complicated mTBI group compared to the uncomplicated group. Although the association may be confounded by injury severity, in the light of our results, the possibility that intracranial bleeding is causally related to fatigue and might be prevented by TXA treatment deserves further consideration.

Tranexamic acid substantially reduces blood loss in surgery ^24^. The CRASH-2 trial ^3^ showed that prompt tranexamic acid treatment reduces death due to bleeding in trauma patients. The CRASH-3 trial showed that early treatment with tranexamic acid reduces head injury deaths in mild and moderately injured TBI patients, potentially by reducing intracranial bleeding ^25^.

Previous studies have shown that tranexamic acid is safe and reduces head injury-related deaths in TBI patients if given within 3 hours of injury. We were unable to demonstrate that tranexamic acid prevents fatigue in mTBI patients. Further randomised controlled trials investigating the effect of early tranexamic acid treatment on disability and death in mTBI patients are needed.

## Data Availability

Individual de-identified patient data from the CRASH-3 trial is available from the The Free Bank of Injury and Emergency Research Data (freeBIRD) website: https://freebird.lshtm.ac.uk/. LSHTM CTU regulations require an account to be created before data can be accessed. Our registration process only requires an email address and takes less than five minutes. The CRASH-3 trial protocol is available at https://freebird.lshtm.ac.uk/. The protocol includes the information patients were given before trial enrolment and also the patient consent form. Zenodo: CONSORT checklist for ‘Effect of early tranexamic acid treatment on fatigue in patients with mild traumatic brain injury: data from the CRASH-3 clinical trial.’ https://doi.org/10.5281/zenodo.5730383 ^13^. Data are available under the terms of the Creative Commons Attribution 4.0 International license (CC-BY 4.0).
