# Supplementary material for: Effect of early tranexamic acid treatment on fatigue in patients with mild traumatic brain injury: data from the CRASH-3 clinical trial
Source: Wellcome Open Res. 2024 Oct 28;6:346. Originally published 2021 Dec 15. [Version 3] doi: 10.12688/wellcomeopenres.17421.3 (PMC11537225; doi:10.12688/wellcomeopenres.17421.3)
Supplement: Peer review history

# Reviewer report: Reviewer response for version 3

**DOI:** 10.21956/wellcomeopenres.25699.r111410

**Published:** 2024-11-19

**Authors:** Igor Prudovsky

**Affiliations:** 1 Maine Medical Center, Scarborough, USA

Mansukhani and colleagues analyzed the data of the CRASH-3 study to determine where the systemic application of tranexamic acid (TXA) early after the traumatic burn injury (TBI) reduces the trauma-related fatigue in patients. TXA is a potent anti-fibrinolytic drug, which reduces trauma-related hemorrhage. CRASH-3 study demonstrated a significant decrease of lethal outcomes after TBI in TXA-treated patients. The present investigation based on the results of CRASH-3 study did not find an effect of TXA on post-traumatic fatigue. This a statistically sound study, although the assessment of fatigue in CRASH-3 was rather subjective. However, the presented results could stimulate further analysis of the effects of TXA in trauma, which appear to transcend anti-fibrinolysis and are known to also include the suppression of systemic inflammation and edema.

Is the work clearly and accurately presented and does it cite the current literature?

Yes

If applicable, is the statistical analysis and its interpretation appropriate?

Yes

Are all the source data underlying the results available to ensure full reproducibility?

Yes

Is the study design appropriate and is the work technically sound?

Yes

Are the conclusions drawn adequately supported by the results?

Yes

Are sufficient details of methods and analysis provided to allow replication by others?

Yes

Reviewer Expertise:

cell and molecular biology of trauma

I confirm that I have read this submission and believe that I have an appropriate level of expertise to confirm that it is of an acceptable scientific standard.

# Reviewer report: Reviewer response for version 3

**DOI:** 10.21956/wellcomeopenres.25699.r108636

**Published:** 2024-11-05

**Authors:** Garima Yadav

**Affiliations:** 1 All India Institute of Medical Sciences, Jodhpur, India

The trial reports on effect of tranexamic acid on fatigue in mTBI patients and the sample size is pretty impressive but the cases of fatigue in the population are low. Hence, the authors have not concluded on its effect in improving fatigue. I am not convinced about the evidence supporting the biological plausibility of the drug effect on fatigue. Despite a well conducted study, it does not add much to clinical practise.

Is the work clearly and accurately presented and does it cite the current literature?

Yes

If applicable, is the statistical analysis and its interpretation appropriate?

I cannot comment. A qualified statistician is required.

Are all the source data underlying the results available to ensure full reproducibility?

Yes

Is the study design appropriate and is the work technically sound?

Yes

Are the conclusions drawn adequately supported by the results?

Yes

Are sufficient details of methods and analysis provided to allow replication by others?

Yes

Reviewer Expertise:

Gynecology

I confirm that I have read this submission and believe that I have an appropriate level of expertise to state that I do not consider it to be of an acceptable scientific standard, for reasons outlined above.

# Response

**Published:** 2024-11-09

**Authors:** Raoul Mansukhani

**Affiliations:** London School of Hygiene & Tropical Medicine, UK

Dear Dr Yadav,

 Thank you for taking the time to review our article. The CRASH-3 trial steering committee included patient groups. These patient groups requested we conduct an analysis of the impact of the trial treatment on fatigue because extreme fatigue is the symptom that patients find particularly troubling. We therefore believe it is necessary to publish these results. Additionally, a criticism of medical research is the publication bias that arises from only publishing positive and statistically significant findings[1]. By publishing these results, we aim to avoid this pitfall.  Regarding the biological plausibility: Saksvik[2] demonstrated an association between intercranial bleeding and fatigue in patients with mild traumatic brain injury (TBI). The CRASH-3 trial demonstrated that tranexamic acid given promptly reduces head injury deaths in patients with mild and moderate TBI[3], probably by reducing intracranial bleeding[4]. It therefore seems resonable to postulate that tranexamic acid might reduce fatigue in patients with mild TBI. Please let us know if you would like us to make any changes. Otherwise, we would appreciate your approval of our article.

 Yours sincerely

 Raoul Mansukhani

 Research fellow in medical statistics

 1. Hopewell S, Loudon K, Clarke MJ, Oxman AD, Dickersin K. Publication bias in clinical trials due to statistical significance or direction of trial results. Cochrane Methodology Review Group, editor. Cochrane Database of Systematic Reviews [Internet]. 2009 Jan 21 [cited 2024 Nov 8];2010(1). http://doi.wiley.com/10.1002/14651858.MR000006.pub3

 2. Saksvik SB, Karaliute M, Kallestad H, Follestad T, Asarnow R, Vik A, et al. The Prevalence and Stability of Sleep-Wake Disturbance and Fatigue throughout the First Year after Mild Traumatic Brain Injury. Journal of Neurotrauma. 2020 Dec 1;37(23):2528–41. 

 3. CRASH-3 trial collaborators. Effects of tranexamic acid on death, disability, vascular occlusive events and other morbidities in patients with acute traumatic brain injury (CRASH-3): a randomised, placebo-controlled trial. The Lancet. 2019 Nov;394(10210):1713–23. 

 4. The CRASH-3 Intracranial Bleeding Mechanistic Study Collaborators. Tranexamic acid in traumatic brain injury: an explanatory study nested within the CRASH-3 trial. Eur J Trauma Emerg Surg. 2021 Feb;47(1):261–8.   

# Reviewer report: Reviewer response for version 3

**DOI:** 10.21956/wellcomeopenres.25699.r108273

**Published:** 2024-11-04

**Authors:** Vincenzo Menditto

**Affiliations:** 1 Azienda Ospedaliero Universitaria delle Marche, Ancona, Italy

I think that the authors have addressed the concerns I raised in my peer review report appropriately.

Is the work clearly and accurately presented and does it cite the current literature?

Partly

If applicable, is the statistical analysis and its interpretation appropriate?

Yes

Are all the source data underlying the results available to ensure full reproducibility?

Yes

Is the study design appropriate and is the work technically sound?

Yes

Are the conclusions drawn adequately supported by the results?

Partly

Are sufficient details of methods and analysis provided to allow replication by others?

Yes

Reviewer Expertise:

Emergency and internal medicine. Mild traumatic brain injury. Pulmonary embolism. Anticoagulant therapy.

I confirm that I have read this submission and believe that I have an appropriate level of expertise to confirm that it is of an acceptable scientific standard.

# Reviewer report: Reviewer response for version 2

**DOI:** 10.21956/wellcomeopenres.25368.r102425

**Published:** 2024-10-15

**Authors:** Vincenzo Menditto

**Affiliations:** 1 Azienda Ospedaliero Universitaria delle Marche, Ancona, Italy

This study examined the effect of early TXA on fatigue and death in patients with a mild traumatic brain and any intracranial bleeding on the CT scan during the CRASH-3 Trial. This focus was not pre specified. The authors didn't use a validated fatigue scale and this outcome was assessed either at discharge or in hospital after 28 days of injury if the patient had not already been discharged. Extreme fatigue or death was reported in 3.3% of TXA treated patients vs 5.1% in the placebo group, but this composite outcome is disproportionately influenced by deaths.

 The conclusions are drawn adequately supported by the results and discussed citing the current literature. However, the major concern is the biological significance of the results. I suggest to add in the discussion some sentences about the biological background of the findings the authors found in this work. Moreover, the authors should explain why they choose a so early outcome's timing. So, pertinent articles would be cited, almost the following article: 

 Vanier C, Santhanam P, Rochester N, Carter L, Lim M, Kilani A, Venkatesh S, Azad S, Knoblauch T, Surti T, Brown C, Sanchez JR, Ma L, Parikh S, Germin L, Fazzini E, Snyder TH. Symptom Persistence Relates to Volume and Asymmetry of the Limbic System after Mild Traumatic Brain Injury. J Clin Med. 2024 Aug 30;13(17):5154. doi: 10.3390/jcm13175154. PMID: 39274367; PMCID: PMC11396354.

 I suggest checking at page 7 the following: “Tranexamic acid substantially reduces blood loss in surgery. The CRASH-2 trial3 showed that prompt tranexamic acid treatment reduces death due to bleeding in trauma patients. The CRASH-3 trial showed that early treatment with tranexamic acid reduces head injury deaths in mild and moderately injured TBI patients, potentially by reducing intracranial bleeding.

 Previous studies have shown that tranexamic acid is safe and reduces head injury-related deaths in TBI patients if given within 3 hours of injury.”

 Perhaps, they didn’t add a lot to the text.

 No other comments.

Is the work clearly and accurately presented and does it cite the current literature?

Partly

If applicable, is the statistical analysis and its interpretation appropriate?

Yes

Are all the source data underlying the results available to ensure full reproducibility?

Yes

Is the study design appropriate and is the work technically sound?

Yes

Are the conclusions drawn adequately supported by the results?

Partly

Are sufficient details of methods and analysis provided to allow replication by others?

Yes

Reviewer Expertise:

Emergency and internal medicine. Mild traumatic brain injury. Pulmonary embolism. Anticoagulant therapy.

I confirm that I have read this submission and believe that I have an appropriate level of expertise to confirm that it is of an acceptable scientific standard, however I have significant reservations, as outlined above.

## References

[1] : Symptom Persistence Relates to Volume and Asymmetry of the Limbic System after Mild Traumatic Brain Injury. 10.3390/jcm13175154 10.3390/jcm13175154 PMC1139635439274367

# Response

**Published:** 2024-10-22

**Authors:** Raoul Mansukhani

**Affiliations:** London School of Hygiene & Tropical Medicine, UK

Dear Dr Menditto Thank you for reviewing our article. We have made the following changes guided by your comments:

 "However, the major concern is the biological significance of the results. I suggest to add in the discussion some sentences about the biological background of the findings the authors found in this work. Moreover, the authors should explain why they choose a so early outcome's timing. So, pertinent articles would be cited, almost the following article: 

 Vanier C, Santhanam P, Rochester N, Carter L, Lim M, Kilani A, Venkatesh S, Azad S, Knoblauch T, Surti T, Brown C, Sanchez JR, Ma L, Parikh S, Germin L, Fazzini E, Snyder TH. Symptom Persistence Relates to Volume and Asymmetry of the Limbic System after Mild Traumatic Brain Injury. J Clin Med. 2024 Aug 30;13(17):5154. doi: 10.3390/jcm13175154. PMID: 39274367; PMCID: PMC11396354."

**Response: **We have added the following to the discussion: 

 The pathophysiology of fatigue after mild TBI is uncertain. Central fatigue after mild TBI may be associated with lesions to specific brain structures, particularly those in the limbic system involved in attention, concentration and executive function. It is hypothesised that deficits in these processes are interpreted as mental fatigue. For example, Vanier at al recently showed that asymmetry of limbic system structures after mild TBI is associated with the persistence of cognitive symptoms. However, if it is proven that tranexamic reduces fatigue after mild TBI this would suggest a role for inflammation and there are some small cohort studies that show increased levels of pro-inflammatory cytokines in patients with severe fatigue after haemorrhagic stroke. We have also added the sentence:

 Although patients in the CRASH-3 trial were only followed to hospital discharge, early fatigue has been shown to predict more long term post-concussive symptoms.  Note the next two sentances in our discussion highlight limitations in our outcome measure.

 "I suggest checking at page 7 the following: “Tranexamic acid substantially reduces blood loss in surgery. The CRASH-2 trial showed that prompt tranexamic acid treatment reduces death due to bleeding in trauma patients. The CRASH-3 trial showed that early treatment with tranexamic acid reduces head injury deaths in mild and moderately injured TBI patients, potentially by reducing intracranial bleeding.

 Previous studies have shown that tranexamic acid is safe and reduces head injury-related deaths in TBI patients if given within 3 hours of injury.”

**Response: **We have consolidate our last two paragraphs into one which now reads: "Tranexamic acid substantially reduces blood loss in surgery. The CRASH-2 trial showed that prompt tranexamic acid treatment reduces death due to bleeding in trauma patients. The CRASH-3 trial showed that early treatment with tranexamic acid is safe and reduces head injury deaths in mild and moderately injured TBI patients, potentially by reducing intracranial bleeding. We were unable to demonstrate that tranexamic acid prevents fatigue in mTBI patients. Further randomised controlled trials investigating the effect of early tranexamic acid treatment on disability and death in mTBI patients are needed." Would you be able to appove our article?

 Yours sincerely

 Raoul Mansukhani

 Research fellow in medical statistic

# Reviewer report: Reviewer response for version 1

**DOI:** 10.21956/wellcomeopenres.19261.r86356

**Published:** 2024-08-12

**Authors:** Christie Fritz

**Affiliations:** 1 Harvard Medical School, Boston, Massachusetts, USA

Thank you for the opportunity to review “Effect of early tranexamic acid treatment on fatigue in patients with mild traumatic brain injury: data from the CRASH-3 clinical trial”. The authors present an original submission in which they evaluated the effect of TXA in TBI patients on fatigue and death. 

 The research behind TXA and fatigue as a surrogate for disability in significant head injuries has not been completely studied and this study attempts to utilize the existing CRASH 3 data and perform a subgroup analysis.

**General comments:**

 I suggest the authors define mTBI in their introduction.

**This study has a few significant issues that are concerning:**

- Outcome measure: fatigue was assessed only by the clinician so isn’t the patient centered outcome that we may necessarily hope for.  Also, fatigue was measured either at hospital discharge (not as useful, since the hospital is a difficult place to measure fatigue at discharge), or at 28 days after injury while still inpatient.  Hospital delirium would appear to be a significant factor in this, and I’m concerned this is not as useful a marker/outcome, especially as a patient centered marker.
- They posit that severely injured patients are more likely to suffer effects from blood and would be less likely to benefit from TXA, but do not supply adequate references to make this assertion.
- I have significant issues with the “composite outcome” of extreme fatigue or death.  The authors didn’t seem to get the results they were looking for with “Extreme fatigue” (only 1.1% of patients, most studies they say cite around 30%) in the GCS greater than or equal to 13 group so they added in death as an endpoint.  There are more (34) patients with death as an outcome than extreme fatigue (10) in the treatment arm and I’m concerned that it is the significance of death, not fatigue that are driving these outcomes, since either alone is not significant.  These seem to be disparate outcomes that shouldn’t be logically grouped together.   
- I’d also say that their discussion “suggesting” that TXA may reduce the risk of extreme fatigue is not borne out.  Their CI crosses 1 and is not statistically significant in either of their analyses.  I was also surprised that their first table, which they note were for patients with GCS of 13 or greater included those with no GCS recorded, which is not well explained.
- They also state “crash 3 trial showed that early treatment with TXA reduces head injury deaths in TBI patients, almost certainly by reducing intracranial bleeding” which may be an overstatement of the conclusions of that paper. 
- My biggest concern is their “composite outcome” of death or extreme fatigue.

Is the work clearly and accurately presented and does it cite the current literature?

Yes

If applicable, is the statistical analysis and its interpretation appropriate?

Partly

Are all the source data underlying the results available to ensure full reproducibility?

Yes

Is the study design appropriate and is the work technically sound?

Partly

Are the conclusions drawn adequately supported by the results?

Partly

Are sufficient details of methods and analysis provided to allow replication by others?

Yes

Reviewer Expertise:

EMS, Emergency Medicine, Protocol Development

I confirm that I have read this submission and believe that I have an appropriate level of expertise to state that I do not consider it to be of an acceptable scientific standard, for reasons outlined above.

# Response

**Published:** 2024-08-27

**Authors:** Raoul Mansukhani

**Affiliations:** London School of Hygiene & Tropical Medicine, UK

Dear Dr Fritz,

 Thank you for your comments which we have used to improve our article. When we were developing the CRASH-3 trial protocol we asked patient groups for their input. Their main suggestion was that we conduct an analysis of the impact of the trial treatment on fatigue because extreme fatigue is the symptom that patients find particularly troubling. We do not want to make any claim that is not supported by the data but we do feel an obligation to publish the results as promised. We accept that we may have overstated the potential of TXA to reduce this outcome.

 Please see below for our responses:    

**Comment 1:** Outcome measure: fatigue was assessed only by the clinician so isn’t the patient centered outcome that we may necessarily hope for. Also, fatigue was measured either at hospital discharge (not as useful, since the hospital is a difficult place to measure fatigue at discharge), or at 28 days after injury while still inpatient. Hospital delirium would appear to be a significant factor in this, and I’m concerned this is not as useful a marker/outcome, especially as a patient centered marker.

**Response:**** We’ve changed the abstract conclusions to read:   Given, 1) our analyses were not prespecified, 2) our outcome measure is not based on a validated fatigue severity scale, and 3) TBI patients can suffer from hospital-induced delirium, which hinders clinician assessment, these results need to be replicated in another study. We have added the following to the discussion: Our analysis was not prespecified, the precision of the estimates was low and our outcome measure is not based on a validated fatigue scale. In addition, TBI patients are prone to hospital-induced delirium which will hinder clinician assessment of fatigue. A larger trial is needed to test the hypothesis that early tranexamic acid treatment reduces fatigue in mTBI patients. ...... The CRASH-3 trial assessed fatigue via a three-point scale which hasn’t been validated. We acknowledge this is a significant limitation with our study. Ideally, fatigue would be measured using a validated scoring system such as the 9 item Fatigue Severity Scale (FSS). We have removed the next sentence: However, studies that use more elaborate scoring systems with long questionnaires tend to have fewer participants and this would increase random error. Misclassification of an outcome variable usually biases towards the null.  

**Comment 2:** They posit that severely injured patients are more likely to suffer effects from blood and would be less likely to benefit from TXA, but do not supply adequate references to make this assertion.

**Response:** *I*n the discussion we have changed the sentence in the discussion: Although we planned to examine the impact of TXA treatment on extreme fatigue, the focus on mild TBI was not pre-specified but stimulated by the observation from the CRASH-3 intracranial bleeding mechanistic study ^17^ that patients with less severe TBI may have less intracranial bleeding at baseline and so there is more potential to prevent bleeding.  

**Comment 3:** I have significant issues with the “composite outcome” of extreme fatigue or death. The authors didn’t seem to get the results they were looking for with “Extreme fatigue” (only 1.1% of patients, most studies they say cite around 30%) in the GCS greater than or equal to 13 group so they added in death as an endpoint. There are more (34) patients with death as an outcome than extreme fatigue (10) in the treatment arm and I’m concerned that it is the significance of death, not fatigue that are driving these outcomes, since either alone is not significant. These seem to be disparate outcomes that shouldn’t be logically grouped together. 

**Response:** We have added the following to our methods: This composite outcome is consistent with the International Council for Harmonisation of Technical Requirements for Pharmaceuticals for Human Use (ICH) E9 guidelines’ "Composite strategy" for handling intercurrent events. We have added the following sentence to the results section of the abstract: This composite outcome is disproportionately influenced by deaths which account for 74% (81 from 110) of events. We added the following sentence to the results section: This composite outcome is disproportionately influenced by deaths which account for 74 % (81 from 110) of events. We have also changed the conclusions of our study based on extreme fatigue alone rather the composite outcome of extreme fatigue and death. (See our response to comment 4 below)  

**Comment 4:** I’d also say that their discussion “suggesting” that TXA may reduce the risk of extreme fatigue is not borne out. Their CI crosses 1 and is not statistically significant in either of their analyses. I was also surprised that their first table, which they note were for patients with GCS of 13 or greater included those with no GCS recorded, which is not well explained. **Response:** We have changed the abstract conclusions to read: We found no evidence that tranexamic acid reduces fatigue in patients with mTBI. We changed the first sentence in our discussion to read:

 Our study found no evidence that tranexamic acid reduced fatigue in patients with mTBI. We replaced the sentence: The results of this study suggest that promptly treating mTBI patients with tranexamic acid may reduce risk of fatigue. With We were unable to demonstrate that tranexamic acid prevents fatigue in mTBI patients.  

**Comment 5:** They also state “crash 3 trial showed that early treatment with TXA reduces head injury deaths in TBI patients, almost certainly by reducing intracranial bleeding” which may be an overstatement of the conclusions of that paper. 

**Response:** We have changed our discussion to read: The CRASH-3 trial showed that early treatment with tranexamic acid reduces head injury deaths in mild and moderately injured TBI patients, potentially by reducing intracranial bleeding.  

**Comment 6:** My biggest concern is their “composite outcome” of death or extreme fatigue.

**Response: **We believe we have addressed this in points 3 and 4 above. I do hope you will  be able to approve our article.  

 Yours Sincerely  

 Raoul Mansukhani

 Research fellow in medical statistics

# Reviewer report: Reviewer response for version 1

**DOI:** 10.21956/wellcomeopenres.19261.r76923

**Published:** 2024-05-11

**Authors:** Kim Kirby

**Affiliations:** 1 University of the West of England,, Bristol, UK

This study sought  to investigate the effect of early tranexamic acid on fatigue in patients  with a mild traumatic brain injury during the CRASH-3 Trial. This focus on fatigue on mild traumatic brain injury was not pre specified. CRASH-3 recruited patients with a GCS less than or equal to 12 or with intracranial bleeding on the CT scan and no significant extracranial bleeding. Patients were randomly allocated to TXA or placebo. In CRASH-3 there were 2,632 patients with a mild traumatic brain injury who had a GCS score of between 13 and 15 and intracranial bleeding on the CT scan and these patients were the focus of this study. Fatigue was assessed and recorded by a clinician and the analysis plan estimated the risk ratio of being in the extreme fatigue category. In the study 4.2% of patients either died, or experienced extreme fatigue. Extreme fatigue was 0.8% TXA v 1.5% placebo.

 This report is well written. It is clearly and accurately presented and easy to understand. The authors adequately address the study limitations (focus on mTBI not prespecified, use of a 3 point scale to measure fatigue, clinician report of fatigue, requirement for a larger adequately powered trial).  I have no revisions to suggest.

Is the work clearly and accurately presented and does it cite the current literature?

Yes

If applicable, is the statistical analysis and its interpretation appropriate?

Yes

Are all the source data underlying the results available to ensure full reproducibility?

Yes

Is the study design appropriate and is the work technically sound?

Yes

Are the conclusions drawn adequately supported by the results?

Yes

Are sufficient details of methods and analysis provided to allow replication by others?

Yes

Reviewer Expertise:

Prehospital research

I confirm that I have read this submission and believe that I have an appropriate level of expertise to confirm that it is of an acceptable scientific standard.
